# The blood serum metabolome profile after different phases of a 4‐km cycling time trial: Secondary analysis of a randomized controlled trial

**DOI:** 10.1002/ejsc.12108

**Published:** 2024-04-23

**Authors:** Rafael A. Azevedo, Ramon Cruz, Marcos D. Silva‐Cavalcante, Adriano E. Lima‐Silva, Romulo Bertuzzi

**Affiliations:** ^1^ School of Physical Education and Sport Endurance Sports Research Group (GEDAE‐USP) University of Sao Paulo Sao Paulo Brazil; ^2^ Faculdade de Medicina FMUSP Applied Physiology and Nutrition Research Group ‐ Center of Lifestyle Medicine Universidade de São Paulo Sao Paulo Brazil; ^3^ Department of Physical Education Sports Center Federal University of Santa Catarina Florianopolis Santa Catarina Brazil; ^4^ Faculty of Nutrition Post‐graduate Program in Nutrition Federal University of Alagoas Maceió Alagoas Brazil; ^5^ Human Performance Research Group Federal University of Technology – Parana Parana Brazil

**Keywords:** bioenergetic, metabolism, nuclear magnetic resonance spectrometry, pacing, self‐paced exercise

## Abstract

It has been assumed that exercise intensity variation throughout a cycling time trial (TT) occurs in alignment of various metabolic changes to prevent premature task failure. However, this assumption is based on target metabolite responses, which limits our understanding of the complex interconnection of metabolic responses during exercise. The current study characterized the metabolomic profile, an untargeted metabolic analysis, after specific phases of a cycling 4‐km TT. Eleven male cyclists performed three separated TTs in a crossover counterbalanced design, which were interrupted at the end of the fast‐start (FS, 600 ± 205 m), even‐pace (EP, 3600 ± 190 m), or end‐spurt (ES, 4000 m) phases. Blood samples were taken before any exercise and 5 min after exercise cessation, and the metabolomic profile characterization was performed using Nuclear Magnetic Resonance metabolomics. Power output (PO) was also continually recorded. There were higher PO values during the FS and ES compared to the EP (all *p* < 0.05), which were accompanied by distinct metabolomic profiles. FS showed high metabolite expression in TCA cycle and its related pathways (e.g., glutamate, citric acid, and valine metabolism); whereas, the EP elicited changes associated with antioxidant effects and oxygen delivery adjustment. Finally, ES was related to pathways involved in NAD turnover and serotonin metabolism. These findings suggest that the specific phases of a cycling TT are accompanied by distinct metabolomic profiles, providing novel insights regarding the relevance of specific metabolic pathways on the process of exercise intensity regulation.

## Highlights

- Untargeted metabolomic profile analysis may reveal unexplored bioenergetic pathways activated throughout a self‐paced cycling time trial.
- Exercise intensity variations throughout a cycling time trial are accompanied by different blood serum metabolomic profiles.
- The current new insights may encourage future investigation on the underpinning mechanisms of specific metabolomic profiles according to each time trial phase.

## 1 INTRODUCTION

Considerable advances in the field of the exercise physiology have been achieved over the past decades by analyzing one (Svedahl & MacIntosh, 2011) or few (Pires et al., 2011) blood metabolites related to the endurance exercise performance. However, most of the previous findings used a targeted metabolite analysis, which precludes a broader view from whole and complex metabolism variance that occurs during endurance exercises (Spriet & Howlett, 1999). In fact, not until recently (Sakaguchi et al., 2019), a targeted metabolite analysis was the only available methodology to investigate metabolism responses to exercise, whereas nowadays analytical platforms that offer integration of several biological databases (i.e., simultaneous measurement of hundreds of metabolites at the same time) have become so available and accessible (Sakaguchi et al., 2019). Indeed, exercise‐induced metabolic responses is a complex interaction of different bioenergetic pathways (Spriet & Howlett, 1999), thus an untargeted investigation could identify and quantify a larger set of metabolites (i.e., metabolome) present in biological tissues and fluids (Zagatto et al., 2022).

Recent studies have utilized different types of endurance‐based exercises (Manaf et al., 2018; Zagatto et al., 2022) to characterize the metabolomic profile. For example, on one hand, an upregulation of blood metabolites related to central nervous system metabolism (i.e., tryptophan) has been suggested to be one of the main causes of task failure in a constant‐load cycling bout of ∼80 min duration at a moderate intensity (Manaf et al., 2018). On the other hand, the metabolomic profile after a constant‐load cycling bout at 115% of maximal aerobic power showed an upregulation in pathways of amino acid and fatty acid oxidation, as well as glycolytic and phosphagen metabolisms (Zagatto et al., 2022). Nevertheless, it must be highlighted that although the topic of exercise intensity effects on metabolomic profile has become a solid line of research, thus leading to publications of systematic reviews on the topic (Kelly et al., 2020; Sakaguchi et al., 2019), there are some caveats that deserve attention. Specifically, the vast majority of the studies that characterized the metabolomic profile in endurance exercises were restricted to constant‐load tasks (i.e., fixed exercise intensity), which has lower ecological validity than self‐paced endurance exercises (Abbiss & Laursen, 2008).

Endurance exercise time trials (TT) are more realistic scenarios of competition (Abbiss & Laursen, 2008) because the exercise intensity is self‐adjusted by the athlete, commonly referred as pacing (Abbiss & Laursen, 2008). Although not explored yet, it is plausible to suppose that pacing directly impacts the metabolic responses because of variations in exercise intensity across domains (Black et al., 2017). In fact, previous study has shown that some physiological responses (i.e., heart rate and oxygen consumption) are attenuated when exercise intensity is self‐regulated throughout an endurance time trial compared to when it is fixed at a constant‐load (i.e., average speed performed from the start to the finish line) (Billat et al., 2006). In a middle‐distance cycling time trials (i.e., 4‐km TT), there is an initial high‐intensity exercise (i.e., fast‐start, FS), followed by a lower and constant exercise intensity (i.e., even‐pace, EP), and a final sprint at the last portion (i.e., end‐spurt, ES). Importantly, whereas the FS and ES phases are often performed within the severe‐intensity domain, the EP phase is performed within the heavy domain (Azevedo et al., 2019; Lima‐Silva et al., 2010; Silva‐Cavalcante et al., 2013). As a result, in FS and ES phases, there is greater anaerobic contribution and metabolic disturbance compared to the EP (Azevedo et al., 2019; Lima‐Silva et al., 2010; Silva‐Cavalcante et al., 2013). It is worth mentioning that the self‐regulation of exercise intensity throughout a TT is a decision‐making process based on sensory inputs (i.e., such as metabolic responses to exercise) and cognitive processes (i.e., assessment of potential risks and feedforward mechanisms). For example, data from our laboratory have shown that exercise intensity variations throughout the TT, such as the FS, EP, and ES phases, possibly, occur in order to adjust the rate of fatigue symptom development, thus avoiding premature exercise cessation and achievement the best performance (Azevedo et al., 2019). However, to date, no study has investigated the metabolome profile after each TT phase, which could reveal new bioenergetic pathways that may be relevant for endurance performance and self‐regulation of exercise intensity throughout the task. Moreover, by characterizing the metabolome profile after each phase of the pacing strategy described above, thus providing a time‐series information, more insights can be gathered regarding the effects of the transient metabolic changes on the process of self‐regulation in exercise intensity throughout the time trial. Therefore, the present study characterized the serum metabolome profiles after specific parts of a 4‐km cycling TT (i.e., FS, EP, and ES phases). We hypothesized that the metabolomic profile would be different after the three main phases of the self‐paced exercise.

## 2 MATERIAL AND METHODS

Eleven male cyclists (age 32 ± 5 years; body mass 77.5 ± 9.4 kg; height 181.0 ± 8.8 cm; maximal heart rate, 180 ± 7 beats·min^−1^; peak power output, 372 ± 37 W; maximal oxygen uptake, 54.2 ± 4.8 mL·kg^−1^ min^−1^) participated in the study, who were classified as recreationally trained cyclists (Pauw et al., 2013). No sample size calculations were because this was an exploratory study with untargeted blood metabolome analysis; thus, it was not possible to select a metabolite in order to calculate the effect size. The participants neither had any known neuromuscular or cardiovascular disorder nor used any medications during their participation in the study. All study procedures, as well as protocol, benefits, and risks were explained before the commencement of trials, and participants gave their written informed consent. The study was conducted according to the Declaration of Helsinki and was approved by the local Research Ethics Committee (process *n*º 111285/2015). Importantly, the present study is part of a larger research project, thus some of the physiological results were not presented here and can be found in a previous publication (Azevedo et al., 2019). Briefly, in that previous publication, we aimed to investigate the neuromuscular fatigue responses throughout the 4‐km TT phases but did not include the metabolomic profile, since this topic deserved an in‐depth analysis and discussion.

### 2.1 Experimental design

**FIGURE 1:**
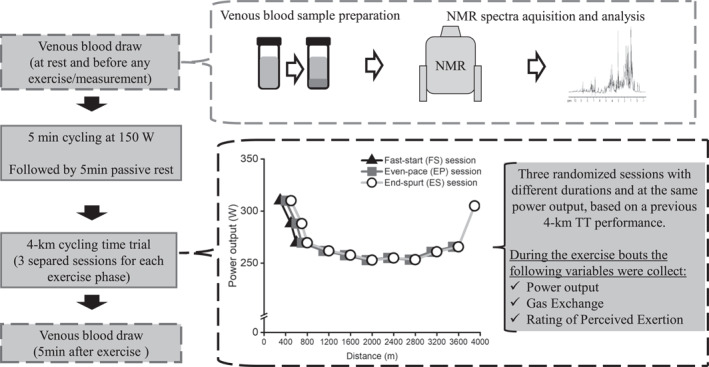
Schematic figure for the experimental sessions. The flow‐chart of an experimental session is represented on the left‐hand side panel. Briefly, before any exercise, venous blood samples were taken at complete rest, which was followed by a 5‐min cycling warm‐up at a PO of 150 W. Thereafter, on separated sessions, the participants cycled for different durations of a pre‐established PO based on the best 4‐km TT performance in the familiarization sessions. As shown in the bottom right panel, the schematic output from a participant is highlighted for the three experimental sessions in different colors and symbols. Thus, the participant replicated the same PO and pacing strategy up to the point which exercise was stopped (i.e., either FS or EP phases' sessions). During the exercise bouts, the anaerobic and aerobic bioenergetic pathways were estimated and the rating of perceived exertion (RPE) was recorded. Finally, 5 min post‐exercise, another venous blood sample was obtained and later processed in the nuclear magnetic resonance (NMR) to obtain the metabolites concentrations. PO, power output.

As shown in Figure 1, the participants visited the laboratory on six separate sessions over a 3‐week period, 48 h apart, and at the same period of the day. In the first session, participants' body mass and stature were measured and a maximal incremental cycling test was performed. In the second and third sessions, participants were familiarized with the 4‐km TT, in which they were instructed to cover the 4‐km distance as fast as possible. Additionally, the power output (PO) distribution (i.e., pacing strategy) from the best 4‐km TT familiarization trial (i.e., shortest time to cover the 4‐km distance) was utilized in the fourth, fifth, and sixth sessions in order to replicate the same exercise intensity variations. Briefly, from the fourth until the sixth sessions, the participants performed, in a randomized fashion, the same PO and pacing strategy as done in their best 4‐km TT familiarization trial but the exercise was interrupted at different time points in the three experimental sessions (i.e., the end of the FS, EP, or ES phase). In order to replicate the same PO in the three experimental sessions (i.e., FS, EP, and EP) the participants were asked to cycle following a pre‐programmed avatar on the cyclosimulator software (RacerMate®, Computrainer™), based on their own PO and pacing strategy from their best 4‐km familiarization TT performance. Importantly, once the exercise was interrupted (i.e., either the FS or EP sessions), the participant did not return to exercise and waited 5 min post‐exertion before the blood sample was collected. Thus, each session was composed by one single exercise bout, which lasted up to either the end of the FS, EP, or ES (i.e., this later session was the full 4‐km TT). These three phases were individually identified by a mathematical method for pacing analysis (Azevedo et al., 2017), which utilizes the mean PO maintained by participants from 25% until 75% of the trial (i.e., Even‐Pace phase, EP) plus two standard deviations to identify the boundaries of the other two phases. The FS phase is determined from the start until the point where the PO decreases be than the set boundaries, whereas the ES phase is determined at the point where the PO rises above the boundaries during the last 75% of the trial. A schematic representation of experimental trials is shown in Figure 1. Blood samples were collected before and 5 min after exercise cessation to acquire their metabolomic profile. Gas exchanges and rating of perceived exertion were also measured throughout the cycling trials. All tests were performed at a constant room temperature (23–24^o^C) and 2 h after the last meal. The participants were instructed to refrain from any exhaustive or unaccustomed exercise that was not related to the study protocol, alcohol and caffeine 48 h before the sessions. A 48‐h food diary was completed before the first session, and participants were instructed to replicate before the remaining sessions.

### 2.2 Preliminary tests

The maximal incremental cycling test was carried on a cyclosimulator (RacerMate®, Seattle), which was calibrated before each test. After a 5‐min warm‐up at 150 W, PO was increased to 30 W·min^−1^ until task failure. Participants exercised at a pedal cadence of 80–90 rpm and task failure was defined as pedal cadence below 80 rpm for 5 s despite strong verbal encouragement. Gas exchange was measured breath‐by‐breath (Cortex Metalyzer 3B) and subsequently averaged over 30 s intervals throughout the test. Heart rate was measured using a transmitter (Polar Electro Oy). The maximal oxygen uptake (V̇O_2_max) was determined as the highest value over 30 s moving average bins. The maximal heart rate was defined as the highest value obtained at the end of the test.

### 2.3 Time trials procedures

The 4‐km TT tests were conducted on the same cyclosimulator, adopting the same warm‐up routine and the same procedures for gas exchange as described above. The distance and PO were recorded at 1 Hz, with the overall performance in each familiarization trial determined as the time expended to cover the 4‐km TT. In the experimental trials, participants were instructed to follow an avatar on the cyclosimulator software, which reproduced the same PO of the best familiarization performance. However, the FS trial was interrupted after the end of the fast start (average distance, 600 ± 205 m), while the EP trial was interrupted after the end of the EP phase (3600 ± 190 m). For the ES trial, participants followed the avatar during the entire 4‐km TT (4000 m). The three phases were identified in accordance with the procedures previously suggested (Azevedo et al., 2017).

### 2.4 Aerobic and anaerobic bioenergetic pathways

The aerobic (P_AER_) and anaerobic (P_ANAE_) power were calculated based on the V̇O_2_ and the respiratory exchange ratio (RER) data during the 5‐min warm‐up and 4‐km TT, according to previous study (Hettinga et al., 2007). Firstly, the gross efficiency was calculated during the 5‐min cycling at 150 W, in which the last 2 min of V̇O_2_ and RER data were utilized to calculate the metabolic power (P_MET_) using the following equation:

$$P_{\text{MET}}\;\left(W\right)=\dot{V}O_{2}\;\left(L\cdot \min^{-1}\right)x\;\left(\left(4940\;\cdot \;\text{RER}+16040\right)/60\right)$$

The gross mechanical efficiency was then obtained by the result of mechanical PO during the warm‐up cycling PO over P_MET_ (i.e., 150 W/P_MET_). For the P_MET_ calculations during the TT, RER was assumed to be equivalent to 1, as previously suggested (de Koning et al., 2013). The P_AER_ was calculated by multiplying the gross mechanical efficiency by P_MET_, and P_ANAE_ was obtained by subtracting the calculated P_AER_ from the mechanical PO (Hettinga et al., 2007). Specifically, the V̇O_2_ data needed to calculate P_MET_ throughout each TT phase, and 5‐min cycling at 150 W, were derived from pulmonary gas exchange measured breath‐by‐breath using a gas analyzer (Cortex Metalyzer 3B, Cortex Biophysik, Leipzig), which was calibrated according to the recommendations of the manufacturer. There were no differences among FS, EP, and ES sessions during the 5‐min cycling at 150 W for V̇O_2_ (2.09 ± 0.16 L·min^−1^, 2.07 ± 0.20 L·min^−1^ and 2.08 ± 0.33 L·min^−1^, respectively; *p* = 0.246), RER (0.88 ± 0.04, 0.88 ± 0.04 and 0.87 ± 0.04, respectively; *p* = 0.933), and gross efficiency (21 ± 2%, 21 ± 2% and 21 ± 3%, respectively; *p* = 0.386).

### 2.5 Metabolomics measurement

#### 2.5.1 Blood samples

A total of 5 mL of blood were collected from the vein of the antecubital fossa at pre and 5‐min post exercise. The 5‐min post exercise delay was chosen because other time‐sensitive procedures were taken before, as highlighted earlier. Blood samples were collected in vacutainer tubes (VACUETTE®, Austria) and were kept at room temperature for 30 min. Then, blood samples were centrifuged at 3000 rpm for 10 min at 4ºC and serum stored at −80ºC for posterior analysis. Before performing the metabolite analysis, the serum samples were filtered through a 3 kDa filter, previously washed to eliminate glycerol. A total of 500 μL of H_2_O Milli‐Q was applied to the 3kDA filter and then centrifuged at 14,000 rpm for 10 min at 4ºC. This procedure was performed five times for total glycerol elimination. After the fifth wash, a rotation cycle with a filter inversion was completed at 8000 rpm for 5 min for residual elimination of Milli‐Q H_2_O. Thereafter, 350 μL of serum was added to the filter and centrifuged at 14,000 rpm for 45 min at four ºC. Then, 300 μL of filtered serum, 60 μL of phosphate buffer solution (1M, pH 7.4) in D_2_O containing 5 mM of TMSP, and 240 μL of H_2_O Milli‐Q were pipetted into a 5 mm NMR tube, producing a final solution with 100 mM phosphate buffer (pH 7.4), 10% D_2_O, and 0.5 mM TMSP.

### 2.6 NMR spectroscopy and data pre‐processing

The NMR spectrums were obtained using Inova Agilent (Agilent Technologies Inc.) and in a resonance frequency of 600 MHz and constant temperature of 298 K (25°C). A total of 256 free induction decays were collected. The spectral phase, baseline correction, identification, and quantification of blood serum sample metabolites were analyzed using Chenomox NMR Suite 7.6 software (Chenomox Inc.).

### 2.7 Statistical analysis

The normal distribution was confirmed by Shapiro–Wilk's test and results were reported as means ± SD. The mean values from PO, P_AER,_ P_ANAE,_ and RPE were compared with repeated measures one‐way ANOVA by using phases as factor (i.e., FS, EP, and ES). When *F* values were significant, Tukey post‐hoc was applied. The metabolomics analysis (Cruz et al., 2021; Xia et al., 2013) were performed as fold change (post/pre ratio), and posteriorly log‐transformed and auto‐scaled to improve approximation to a Gaussian distribution. In order to describe and summarize the metabolomic differences between phases, a principal component analysis (PCA) was conducted, which reduces the dimension of a large dataset and facilitates its understanding. Thereafter, in order to identify which metabolites were discriminating the metabolomic profile between phases, a supervised Partial Least Square–Discriminant Analysis (PLS‐DA) was conducted. The PLS‐DA analysis was validated by the following criteria: i) analysis of the coefficient of determination—R^2^; ii) evaluation of the predictive capacity of the model (Q^2^, considering the valid models with >0.40); and iii) permutation test calculations in 1000 permutations. From the PLS‐DA results, the variable importance in the projection (VIP) was used to indicate which metabolites were responsible for discriminant analysis. Finally, the metabolites with VIP values > 1.00 were considered for Enrichment Analysis (EA), which identifies possible different metabolic pathways between phrases and interpret pattern based on a group of metabolite instead individual analysis. Valid metabolic pathways were considered when statistically significant (i.e., *p*‐value <0.05) and at least two associated metabolites (i.e., hits). Additionally, the false discovery rate (FDR) was reported for each metabolic pathway. The associated metabolites to each pathway were verified based on the “Homo Sapiens” SMPDB and KEGG libraries. Significance level was set at *p* ≤ 0.05. All statistical analyses were performed using a statistical software package (SPSS, version 13.0). All metabolomic analyses were processed using the MetaboAnalyst 3.0® software.

## 3 RESULTS

### 3.1 The 4‐km time trial

**FIGURE 2:**
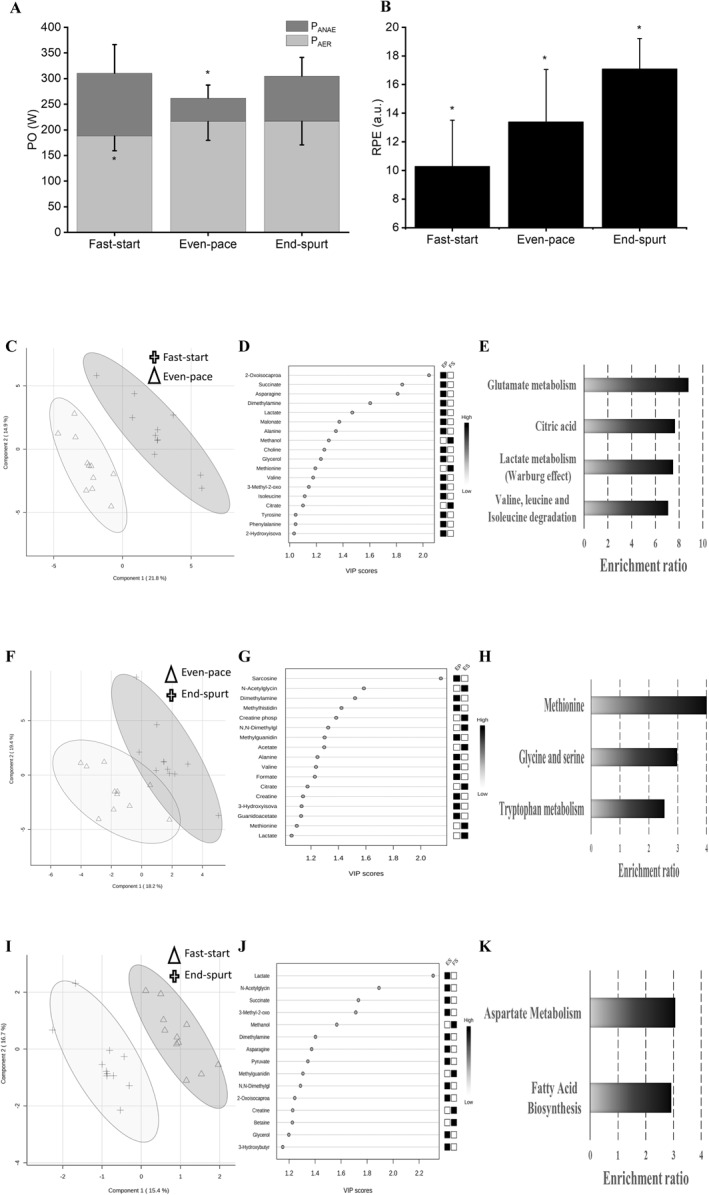
Variables measured at each 4‐km time trial phase. Panel (A) total power output produced in terms of the sum from aerobic (P_AER_) and anaerobic (P_ANAE_) metabolic power throughout each 4‐km time trial phase; Panel (B) RPE throughout each 4‐km time trial phase. Data presented as mean ± SD from each phase of the time trial, as follows: FS, EP and ES. * statistically different from the other phases (*p* < 0.05); Panel (C–E) the PLS‐DA, VIP of each metabolite that discriminate the differences between phases and EA)highlighting the main bioenergetics pathways that differentiates FS and EP phases; Panel (F–H), the PLS‐DA, VIP of each metabolite that discriminate the differences between phases and EA highlighting the main bioenergetics pathways that differentiates EP and ES phases; Panel (I–K) the PLS‐DA, VIP of each metabolite that discriminate the differences between phases and EA, highlighting the main bioenergetic pathways that differentiates FS and ES phases. RPE, rating of perceived exertion; FS, follows: fast‐start; EP, even‐pace; ES, end‐spurt; PLS‐DA, partial least square–discriminant analysis; VIP, variable importance in the projection; EA, enrichment analysis.

In average, the time and distance to cover the FS (63 ± 21 s and 600 ± 205 m), EP (284 ± 40 s and 3600 ± 190 m), and ES (36 ± 19 s and 376 ± 190 m) were different for each participant because the PO and pacing strategy were specific and individualized based on the participant's best 4‐km TT familiarization trial. The total time to complete the 4‐km TT was 382 ± 18 s (i.e., during the ES session the participant cycled the entire 4‐km TT, see Figure 1) and the PO distribution showed a classical “*U*‐shaped” profile which was different among phases (*p* = 0.005). There were higher PO values for FS (311 ± 66 W) and ES (305 ± 44 W) than in EP phase (262 ± 44 W) (*p* = 0.007 and 0.019, respectively); however, there was no difference between FS and ES (*p* = 0.893). P_AER_ was different among phases (*p* < 0.001) (Figure 2A), as there were lower values for FS (62 ± 12%) when compared with EP (83 ± 9%) and ES (72 ± 16%) (*p* = 0.002 and 0.036, respectively), but without difference between EP and ES (*p* = 0.088). P_ANAE_ was also different among phases (*p* < 0.001), as there were higher values in FS (38 ± 12%) and ES (28 ± 16%) than in the EP (17 ± 9%) (*p* = 0.002 and 0.020, respectively), but there was no difference between FS and ES phases for P_ANAE_ (*p* = 0.059). RPE was different among phases (*p* < 0.001, Figure 2B), wherein RPE values increased from FS to ES (all *p* < 0.05).

### 3.2 Metabolomic profile

Based on ^1^H NMR analysis from the serum metabolome, 54 metabolites were identified and quantified (i.e., Figshare Digital Repository; https://doi.org/10.6084/m9.figshare.21391134.v3). Overall, the average and range of fold‐changes for the 54 metabolite identifies were similar among the FS (average, 1.28; range, 0.16–25.32), EP (average, 1.39; range, 0.07–13.80) and ES (average, 1.44; range, 0.10–24.28) phases.

For the FS versus EP phase comparison, there was a clear distinction between phases (Figure 2C). The PLS‐DA analysis validation tests indicated high values of *R* ^2^ = 0.99, *Q* ^2^ = 0.81, and permutation = 0.003. A total of 18 metabolites showed VIP scores > 1.00 (Figure 2D), wherein all of them increased from the FS to EP phase (i.e., see Figshare Digital Repository; https://doi.org/10.6084/m9.figshare.21391134.v3). The enrichment analysis (EA) between the FS and EP phases showed that four metabolic pathways were more expressed, as shown in Figure 2E and Table 1.

**TABLE 1:** Summary of the significantly enriched pathways between phases throughout the 4‐km cycling TT.

Pathways	Library	*p*‐value	FDR	Enrichment ratio	Match status	Hits
FS and EP phases						
Glutamate metabolism	SMPDB	0.009	0.001	8.812	2/49	Alanine; Succinate
	KEGG					
Citric acid	SMPDB	0.005	0.0007	7.667	2/32	Citrate; Succinate
	KEGG					
Warburg effect	SMPDB	0.0009	0.0009	7.522	3/58	Citrate; Lactate; Succinate
Valine, leucine, and isoleucine metabolism	SMPDB	0.0004	0.0001	7.092	5/60	2‐Oxoisocaproate; Isoleucine; Succinate; Valine
	KEGG					
EP and ES phases						
Methionine	SMPDB	0.003	0.121	4.034	3/43	N,N‐Dimethylglycine; Methionine; Sarcosine
	KEGG					
Glycine and serine	SMPDB	0.011	0.184	2.981	6/59	Alanine; Creatine; N,N‐Dimethylglycine; Guanidoacetate; Methionine; Sarcosine
	KEGG					
Tryptophan	SMPDB	0.047	0.232	2.548	2/60	Alanine; Formate
	KEGG					
FS and ES phases						
Aspartate Metabolism	SMPDB	0.049	0.18	3.062	2/35	Acetate; Asparagine
	KEGG					
Fatty Acid biosynthesis	SMPDB	0.042	0.8	2.915	2/35	Acetate; 3‐Hydroxybutyrate
	KEGG					

*Note*: Data presented as mean ± SD. Values herein presented as fold change from each session (i.e., post/pre‐exercise ratio).

For EP versus ES phases comparison of metabolic profiles (Figure 2F), the PLS‐DA analysis validation tests indicated high values of *R* ^2^ = 0.98, *Q* ^2^ = 0.71, and permutation = 0.02. A total of 17 metabolites showed VIP scores > 1.00 (Figure 2G) (i.e., Figshare Digital Repository; https://doi.org/10.6084/m9.figshare.21391134.v3). Additionally, the EA between the EP and ES phases showed three metabolic pathways more expressed, as shown in Figure 2H and Table 1.

The FS versus ES phases comparison of metabolic profiles (Figure 2I), the PLS‐DA analysis validation tests indicated high values of *R* ^2^ = 0.99, *Q* ^2^ = 0.76, and permutation = 0.048. A total of 15 metabolites showed VIP scores > 1.00 (Figure 2J) (i.e., Figshare Digital Repository; https://doi.org/10.6084/m9.figshare.21391134.v3). Finally, according to the EA analysis between the FS and ES phases, there were two metabolic pathways that were more expressed, as shown in Figure 2K and Table 1.

Additionally, the 48‐h food diary before the three experimental sessions (i.e., FS, EP, and ES) revealed that, in average, the participants consumed a total of 2931.7 ± 183.8 kcal, where 408.4 ± 9.4 g were carbohydrates, 90.60 ± 7.5 g were fat, and 137.6 ± 19.1 g were proteins.

## 4 DISCUSSION

The main findings of this study revealed that: (i) the high exercise intensity adopted during the FS was associated with lower concentration of metabolites involved in the TCA cycle, and its related pathways, compared to EP; (ii) EP showed changes associated with anti‐oxidant effects and oxygen delivery adjustments, whereas ES was related to pathways involved in NAD and serotonin metabolism; (iii) despite the similarities in PO production between FS and ES, the later phase relied more on oxidative phosphorylation contribution, possibily because of the activation of fatty acids and aspartate related pathways. Therefore, by combining the metabolome profile after each pacing strategy phase of a 4‐km cycling time trial, it can be speculated that specific metabolic pathways, which were not previously investigated by target analysis, might influence the process of exercise intensity self‐regulation until the finish line.

### 4.1 The fast‐start and even‐pace phases

The comparison between the metabolomic profiles after the cessation of FS compared to the EP phase was characterized differences in the activation of pathways associated with the production of TCA intermediates (Gibala et al., 2002; Sahlin et al., 1990), such as glutamate, citric acid, and valine metabolisms, as well as pathways associated to lactate production and oxidation (Brooks, 2009). These distinct metabolomic profiles between phases might have been influenced by greater anaerobic metabolism contribution during the FS phase, whereas the aerobic metabolism was more activated during the EP phase. Indeed, previous findings (Hettinga et al., 2006; Silva‐Cavalcante et al., 2013) that utilized the same methodological approach to estimate the contributions of bioenergetic systems showed similar results to the current findings, as well as the overall pacing profile, throughout a 4‐km cycling TT.

The activation of TCA is accompanied, but not limited (Gibala et al., 2002), by the biosynthesis of its intermediates (Sahlin et al., 1990). For example, previous findings showed an increase in TCA intermediates after 5, 10, and 15 min of high‐intensity exercise (Gibala et al., 2002), wherein an anaplerosis effect might occur due to a net increase in carbon influx into the cycle (Gibala et al., 1997). Thus, during the FS phase, the TCA cycle and its related pathways (e.g., glutamate, citric acid, and valine metabolisms) were delayed in its activation and did not reach the full production capacity of TCA intermediates. Additionally, there were also changes related to lactate metabolism, as shown by metabolites associates to the Warburg effect (Brooks, 2009). Even though the Warburg effect is mostly associated with carcinogenic cell metabolisms (Brooks, 2009), lactate production and its oxidation might have occurred in the FS phase and followed throughout the EP, in order to maintain a high exercise intensity (Svedahl & MacIntosh, 2011). It could be suggested that the high exercise intensity and reliance on anaerobic metabolism throughout the FS phase was followed by lower exercise intensity, which elicited activation of aerobic metabolism in the EP phase.

### 4.2 The even‐pace and end‐spurt phases

The comparison between the metabolomic profile after the EP and ES phases showed greater activation of anaerobic metabolism due to the high exercise intensity adopted at the final sprint. Specifically, the enrichment analysis showed changes in the methionine, glycine, and tryptophan metabolisms between EP and ES phases. The methionine and glycine metabolisms are related to the production of glutathione, which is a major systemic antioxidant substance activated by the exercise‐induced production of reactive oxygen species (ROS) (Glynn et al., 2015; Imenshahidi & Hossenzadeh, 2022; Olsen et al., 2020). Additionally, glycine metabolism is also involved in the blood pressure regulation by increasing nitric oxide levels within the vessels (Hafidi et al., 2006), which eliminates the excess of acyl groups derived from branched‐chain amino acids metabolism and muscle damage (Glynn et al., 2015). Thus, the exercise‐induced changes throughout the EP phase might have been related to oxidative phosphorylation metabolism, such as production of ROS, blood vessels tonus regulation, and branched‐chain amino acids metabolism to support the TCA and effects of muscle damage. On the other hand, the higher exercise‐intensity and anaerobic contribution in the ES compared to the EP induced changes in the tryptophan metabolism, which is a precursor of NAD and NADH via kynurenine pathway (Strasser et al., 2016) and serotonin biosynthesis within the central nervous system (Newsholme & Blomstrand, 1996). For example, there is a decline in free‐tryptophan within the blood stream after a 20‐min cycling time trial, probably associated with greater kynurenine levels and diminished serotonin biosynthesis (Strasser et al., 2016). Together, the differences in metabolomic profiles suggest that while the changes in EP were associated with anti‐oxidant effects and oxygen delivery adjustment, the ES changes were related to NAD and serotonin metabolism.

### 4.3 The similarities and differences between the fast‐start and end‐spurt phases

As an exploratory analysis, the comparison between the FS and ES post‐phase metabolomic profiles revealed that, despite similar PO production in these two TT phases, there was a greater contribution from oxidative phosphorylation pathways in the ES compared to the FS phase (i.e., Figure 2A). Specifically, there were distinct metabolomic profiles between phases (i.e., Figure 2I), in which the enrichment analysis highlighted that aspartate and fatty acid metabolisms were more activated in the ES phase compared to the FS phase (i.e., Figure 2K). In fact, the main metabolite hits related to those pathways (i.e., acetate and asparagine; see Table 1 for details) have already been described as relevant biomarkers for endurance performance in rodents (Marquezi et al., 2003; Pan et al., 2015). Thus, it could be suggested that despite the similarities in exercise intensity performed between the FS and ES phases, the underpinning metabolic metabolisms are different since the oxidative phosphorylation, specifically through aspartate and fatty acid metabolism, seems to be more relevant in the later phase than at the beginning of the TT.

### 4.4 Exercise intensity variations and metabolomic profiles

This is the first study to characterize, in a time‐series fashion, the metabolomics profile after each phase of a self‐paced cycling, which might provide valuable insights regarding the mechanisms underpinning exercise‐intensity variations throughout the exercise. It must be highlighted that in comparison to the literature on the topic of exercise intensity and its effects on metabolomic profile (Sakaguchi et al., 2019), the 4‐km TT phases elicited an average fold‐change in metabolic responses slightly lower (i.e., fold‐change, ≥2.0) than in other studies (Manaf et al., 2018; Nieman et al., 2014) that utilized constant‐load endurance exercise tasks performed at high‐intensity (i.e., within the heavy‐domain exercise intensity) and long‐duration (i.e., ≥60 min). However, the metabolic pathways that were found to be highly activated in those studies were similar to the current results, such as lipid metabolism, activation of TCA cycle, and/or glycolytic‐related metabolites, in which this last metabolic response was mostly found in moderate intensity (i.e., 55% of V̇O_2_max) and short‐duration (i.e., ∼30 min) exercise bouts (Jacobs et al., 2014; Sakaguchi et al., 2019). Thus, it could be suggested that the time‐series of the metabolomic responses after each phase of the cycling TT was of similar magnitude compared to constant‐load endurance exercise bouts. Importantly, the current findings further expand the understanding about the interconnection between exercise intensity variations and metabolic responses throughout a self‐paced cycling TT, mostly because the metabolomic profiles were acquired specifically after key change‐points of the pacing strategy adopted from each individual.

Self‐paced endurance exercises involve a complex decision‐making process compared to constant‐load tasks (Renfree et al., 2014), since exercise‐intensity variations involves several internal (i.e., physiological and/or perceptual responses) and external signals (i.e., environment clues) in order to avoid premature exercise cessation (Noakes, 2012). Our results showed that RPE values consistently increased throughout the 4‐km TT, while there were considerable variances in exercise intensity and post‐exercise metabolomic profile. The untargeted analysis utilized provided insights of possible relevant metabolic pathways that might had interfered in the conscious decision of exercise‐intensity variations, which were not possible with reductionist targeted‐metabolite analysis (Abbiss & Laursen, 2005; Pires et al., 2011). It could be suggested that while the exercise intensity regulation in the FS phase is mostly based on metabolic signals related to the depletion of anaerobic resources, the EP phase might be underpinned by the accumulation of metabolites related to the TCA cycle and oxygen delivery to the working muscles. Additionally, the EP to ES transition (i.e., characterized by an abrupt increase in exercise‐intensity) might have been related to the accumulation of metabolites associated with, but not limited to, the central nervous system function, such as tryptophan pathway and/or ROS accumulation within the muscles (Amann et al., 2011; Newsholme & Blomstrand, 1996; Noakes, 2012). Finally, the exploratory analysis comparing the FS and ES post‐phase metabolomic profile suggested that despite the similar PO performed in those phases, the ES phase might be underpinned by certain oxidative phosphorylation pathways, such as aspartate and fatty acid metabolisms. Nonetheless, the present findings highlight the complex interconnection among exercise intensity variations, metabolic changes, and RPE responses at each phase of a cycling TT.

### 4.5 Limitations

First, the present study design assessed the metabolomic profile after 5 min from exercise cessation rather than during and/or immediately after, mostly because of other procedures that have been made immediately after the exercise cessation, which were presented in previous publication from the current dataset (Azevedo et al., 2019). As a result, the metabolomic profile post each TT phase may not represent the metabolic responses as seen during and/or immediately after exercise cessation. In fact, the metabolomic profile is somewhat different when comparing immediately post versus 20 min after exercise cessation (Manaf et al., 2018), but still different from the baseline metabolomic profile. Nonetheless, the advantages of the current methodological approach are: (i) self‐selected exercise intensity, thus pacing, is not disrupted by blood draw procedures during the exercise, since it has been extensively shown that environmental and/or internal factor affects TT pacing strategy and performance (Abbiss & Laursen, 2005; Noakes, 2012); (ii) 5‐min post‐exercise cessation metabolomic assessments may allow enough time for the key metabolism by‐products to reach the blood stream, such as occurs in delayed blood lactate levels rise after high‐intensity exercise bouts (i.e., highest levels of blood lactate around 7.5 min post‐exercise) (Vandewalle et al., 1987); and (iii) it was possible to gather information regarding the transient nature of the metabolomic profile and its possible relation to self‐regulated exercise intensity because the blood samples were taken after key points of the pacing strategy from each individual. Finally, our experimental design did not include a comparison between self‐paced versus constant‐load exercises at the same PO and duration of each TT phases (i.e., FS, EP, and ES), which could provide more insights regarding the actual effect of exercise intensity variations per se in the metabolomic profile at a matched PO and duration exercise bouts, also allowing further comparison with previous literature that utilized constant‐load cycling bouts (Sakaguchi et al., 2019).

## 5 CONCLUSION

The current findings showed, for the first time, the transient characteristic of the metabolome profile throughout a self‐paced cycling time trial, since it was found distinct metabolomic profiles after specific phases of a 4‐km cycling time trial. The post‐exercise metabolomic profile differences between the first and middle phases were mostly related to the accumulation of metabolites involved in the anaerobic metabolism and the activation of aerobic pathway, as well as metabolic systems related to oxygen delivery to the active musculature. Additionally, the difference between the middle and the end phases was characterized by metabolic pathways related to central nervous system functioning and oxidative stress. Finally, despite similar PO production, and exercise intensity, between the very beginning and last meters of the 4‐km time trial, the energy production in the latter phase was mostly derived from oxidative phosphorylation pathways.

## CONFLICT OF INTEREST STATEMENT

The authors report that there are no competing interests to declare.
